# Recent developments and emerging trends of mass spectrometric methods in plant hormone analysis: a review

**DOI:** 10.1186/s13007-020-00595-4

**Published:** 2020-04-16

**Authors:** Liyuan Wang, Yilin Zou, Han Yeong Kaw, Gang Wang, Huaze Sun, Long Cai, Chengyu Li, Long-Yue Meng, Donghao Li

**Affiliations:** 1 Department of Chemistry, MOE Key Laboratory of Biological Resources of the Changbai Mountain and Functional Molecules, Yanbian University, Park Road 977, Yanji, 133002 China; 2 Department of Environmental Science, Yanbian University, Yanji, 133002 China; 3 State Key Laboratory of Application of Rare Earth Resources, Changchun Institute of Applied Chemistry, Chinese Academy of Sciences, Changchun, 130022 China

**Keywords:** Plant, Plant hormone, Sample pretreatment, Mass spectrometry, Trace analysis

## Abstract

Plant hormones are naturally occurring small molecule compounds which are present at trace amounts in plant. They play a pivotal role in the regulation of plant growth. The biological activity of plant hormones depends on their concentrations in the plant, thus, accurate determination of plant hormone is paramount. However, the complex plant matrix, wide polarity range and low concentration of plant hormones are the main hindrances to effective analyses of plant hormone even when state-of-the-art analytical techniques are employed. These factors substantially influence the accuracy of analytical results. So far, significant progress has been realized in the analysis of plant hormones, particularly in sample pretreatment techniques and mass spectrometric methods. This review describes the classic extraction and modern microextraction techniques used to analyze plant hormone. Advancements in solid phase microextraction (SPME) methods have been driven by the ever-increasing requirement for dynamic and in vivo identification of the spatial distribution of plant hormones in real-life plant samples, which would contribute greatly to the burgeoning field of plant hormone investigation. In this review, we describe advances in various aspects of mass spectrometry methods. Many fragmentation patterns are analyzed to provide the theoretical basis for the establishment of a mass spectral database for the analysis of plant hormones. We hope to provide a technical guide for further discovery of new plant hormones. More than 140 research studies on plant hormone published in the past decade are reviewed, with a particular emphasis on the recent advances in mass spectrometry and sample pretreatment techniques in the analysis of plant hormone. The potential progress for further research in plant hormones analysis is also highlighted.

## Background

Plant hormones are small organic molecules that naturally occurring in plants at very low concentrations. They regulate plants germination, growth, reproduction as well as both biotic and abiotic stress responses under different environmental conditions. These molecules show diverse chemical properties and unique chemical structures with wide polarity range and poor photo-thermal stability [1–12]. Plant hormones are categorized into several classes based on their structures and physiological functions [10, 13]. The most common classes are: auxin, cytokinin (CK), abscisic acid (ABA), gibberellin (GA), brassinosteroids (BR), salicylic acid (SA), jasmonic acid (JA), ethylene (ET) and some newly identified plant hormones such as strigolactone (SL) [12]. Each class is defined by its physiological functions in regulating plant growth through synergistic or antagonistic [14, 15]. Therefore, simultaneous quantification of the multi-class plant hormones and accurate determination of spatial–temporal distribution is important to clarify the mechanisms of their recognition, regulation, and how they influence plant growth and development.

Accurate determination of plant hormones requires the development of highly sensitive and efficient analytical techniques for analysis of plant hormone. Effective analysis of plant hormones is limited by inefficient extraction procedures due to their extremely low abundance (usually at ng g^−1^ or even pg g^−1^ level) and varied concentration ranges in plants [16]. For instance, the concentration of auxin and JA in plants ranges about 1–50 ng g^−1^ fresh weight (FW) whereas the content of BRs is as low as 0.01–0.1 ng g^−1^ FW [17]. This calls for highly sensitive analytical strategies with a good dynamic range for quantitative analysis are required for simultaneous extraction and purification of multi-class plant hormones. Existence of several structural isomers of plant hormones also complicates the separation processes. Another factor that affects analysis of plant hormone is the matrix effect. Elimination of interference is important for obtaining accurate information on the metabolism and functions of plant hormones [18]. In addition to these features, plant hormones are unstable and extremely sensitive to the environment change such as temperature, humidity and light due to their chemical heterogeneity. For instance, GAs show high sensitivity to pH and temperature (above 40 °C) but are relatively stable under acidic conditions [19].

These challenges necessitate the development of sample pretreatment and detection methods that are highly selective, sensitive, good enrichment, ease of handling and with broader dynamic range of detection. Non-chromatographic analytical methods such as bioassays and immunoassays were widely used in the early stage of plant hormone research. In comparison with mass spectrometry (MS)-based methodologies, these methods are less sensitive, specific and cannot perform simultaneous detection of multi-class plant hormones, thus, they have been phased out with modern approaches [20]. MS detection equipped with chromatographic techniques are the most widely used analytical approaches for simultaneous analysis of multi-class plant hormones are based on MS detection equipped with chromatographic techniques. Chromatographic methods integrated with MS system provide effective analysis for multi-class plant hormones in the plant matrix (plant component except for plant hormone) [20–23]. Particularly, combining gas chromatography (GC) or liquid chromatography (LC) with different MS detectors forms a rapid, sensitive and high-throughput method for quantitative analysis of plant hormone [21, 24–26]. Furthermore, in order to simultaneous analysis multi-class plant hormones in a single step, a derivatization step is often required before MS-based instrumental analysis because of the non-volatile and non-ionizable property of some plant hormones [27–32]. Thus, derivatization step may promotes comprehensive investigation of the distribution of plant hormone among plant tissues, and hence characterization of metabolic and signaling processes involving plant hormones [33].

MS is a promising technique for efficient analysis of plant hormones. This is because it has high detection performance [1, 11, 33–36]. For this reason, MS-based methods with better sensitivity, selectivity, accuracy and high-throughput features are used to quantitatively and qualitatively analyze plant hormone at tissue and cellular level [29, 37–39]. Given its effective mass-to-charge (m/z) separation ability, mass analyzer provides enhanced selectivity of analytes, thereby improving quantification of trace and ultra-trace plant hormones in plant. Several types of mass analyzers have been developed such as ion trap (IT) [40], quadrupole time-of-flight (Q-TOF) [41] and triple quadrupole instruments (QQQ) [42]. Mass analyzer combined with chromatography have been extensively applied in plant hormone analysis, indicating their ability of accurate quantitative analysis and efficient separation capabilities [43]. MS-based methods can facilitate simultaneous analysis of multiple target compounds. It is therefore suitable for comprehensive analysis and elucidation of the biosynthesis, transportation, metabolic pathways, and signaling networks of plant hormones.

This review provides an up-to-date overview of modern analytical methods and sample pretreatment techniques for plant hormone analysis. Some recent applications of these methods are highlighted in this review. The fragmentation patterns of plant hormones are investigated to provide a theoretical basis for the establishment of a mass spectral database. The prospects related to the analysis of plant hormone are also proposed, which would serve as a technical guide for further discovery of new plant hormones [44, 45].

## Sample pretreatment

The key sample pretreatment strategies applied in plant hormone analysis include homogenization, extraction and purification steps (some studies incorporate a derivatization or labeling step depending on the analytical strategies and instrumental requirements) [42, 43, 46–50]. Sample pretreatment procedure is the major bottleneck that limits rapid separation and simultaneous analysis of multi-class plant hormones, especially at trace levels. To effectively extract plant hormone from complex plant samples, analytical methods that minimize matrix interferences and meet the requirements of high sensitivity, satisfactory recovery and good reproducibility with simple operation are desirable [1, 19, 23, 36, 40, 49–52]. Previous studies have indicated that sample pretreatment is the most labor-intensive and time-consuming step in the whole analytical procedure of endogenous plant hormones [43]. In addition, sample pretreatment is the most error-prone part of the process accounting for about 30% of all sources of errors [10, 13, 18, 43]. An ideal sample pretreatment technique should be less sample consuming, exhibit high sensitivity, and display outstanding enrichment capabilities, especially during in vivo detection.

Accurate determination of variations in the spatial distributions of plant hormone with the aspects of in vivo analysis them in a trace amount of plant tissues would broadening the scope of investigating their physiological functions. The extremely low concentration of plant hormone, similarity in chemical structures and large amount of metabolites in plants are the main challenges which affect accurate detection of plant hormone as they cause interference during analyses [53]. A trace amount of sample is sufficient for investigating the content and dynamic changes of plant hormones in a specific part of the plant. This limits the use of rare and endangered plants as source of sample for analytical purposes. However, only few pretreatment methods are sensitive enough and provide high enrichment capabilities for in vivo analysis of trace amount of plant hormones in plant matrix [1, 51, 53–55]. On-line analysis methods provide a platform for analysis of trace compounds analysis [56]. However, due to the complexity of plant sample matrix and the low concentration of components to be measured, development of on-line techniques has been relatively very slow. In addition to in vivo, in situ, real-time and on-line analysis, sample pretreatment techniques are expected to be highly sensitive with good enrichment properties to produce reliable analytical results for plant samples with high complexity. Typical examples of sample pretreatment methods published in the recent past 5 years are shown in Table 1.

**Table 1:** Representative sample pretreatment methods for the determination of plant hormone

Analytes	Sample pretreatment^a^		Plant matrix	On-/off-line extraction	References
	Extraction solvent	Purification method			
BRs	80% methanol	Solid phase extraction (SPE)	*A. thaliana* leaf (10 g FW)	Off-line	[31]
IAA, ABA, JA, GAs	Acetonitrile	Sequential magnetic solid-phase extraction (MSPE)	*Brassica napus* L. flowers (100 mg FW)	Off-line	[16]
BRs	Methanol	matrix solid-phase dispersion (MSPD)	Rice (200 mg FW)	On-line	[37]
CKs, ABA, GAs, JAs, SA, BRs, SLs	2-proponal/H_2_O/HCl (2:1:0.002 v/v/v)	Solid phase extraction (SPE)	*P. pinaster* Aiton. clones needles (200 ± 20 mg DW)	Off-line	[54]
BRs	Acetonitrile	two-dimensional microscale solid phase extraction (2DμSPE)	Tomato leaves (225 mg FW)	On-line	[57]
SA, IAA, ABA	Methanol	ion-pair stir bar sorptive extraction (IP-SBSE)	Cucumbers and green bean sprouts (100 mg FW)	Off-line	[1]
GAs	75% methanol, 5% formic acid	Liquid–liquid extraction (LLE)	*A. thaliana* flower	Off-line	[21]
GAs, ABA, ET, SAs, JAs, BRs	Methanol: water: HCl (6 N) (80: 19.9:0.1; v/v/v)	Liquid–liquid extraction (LLE)	Valencia sweet orange (100 ± 2 mg FW)	Off-line	[30]
SA ABA	Methanol–water–acetic acid extractionsolution (80:19:1, v/v/v)	Dispersive liquid–liquid microextraction (DLLME)	Peach (250 mg FW)	Off-line	[40]
JA, IAA, SA, ABA, IBA, GA_3_	Centrifugation at 9500 rpm	Single-drop liquid–liquid–liquid microextraction (SD-LLLME)	Fresh fruit juice	Off-line	[58]
ABA, IAA	80% Methanol	Hollow-fiber liquid-phase micro-extraction (HF-LPME)	Soil sample (10 g)	Off-line	[59]
BRs	Acetonitrile	Polymer monolith microextraction (PMME)	Rice shoots (1 g FW leaves, or 0.5 g FW flower tissue)	Off-line	[60]
28-EpihomoBR	80% methanol	Solid-phase microextraction (SPME)	*A. thaliana* samples (400 mg FW)	On-line	[34]
NAA, 2-NOA, 2,4-D, MCPA, PAA	H_2_O,NaCl,HCl	Solid-phase microextraction (SPME)	Tomato (3 g FW)	Off-line	[61]

^a^On-line means coupling of preparation techniques, both for extraction and clean-up, and injection in a detection system; off-line means preparation techniques, both for extraction and clean-up, and injection in a detection system are carried out independently

### Common pretreatment methods in plant hormone analysis

**Fig. 1:**
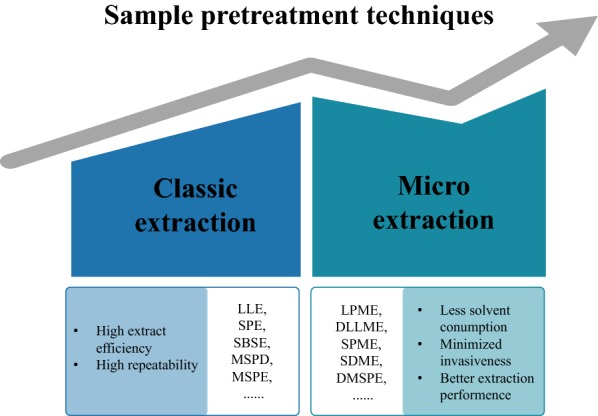
Sample pretreatment methods for the determination of plant hormone. Sample pretreatment methods: LLE liquid–liquid extraction, *LPME* liquid phase microextraction, *DLLME* dispersive liquid–liquid microextraction, *SPE* solid phase extraction, *SPME* solid phase microextraction, *MSPE* magnetic solid phase extraction, *MSPD* matrix solid-phase dispersion, *SDSE* single drop microextraction, *SBSE* stir bar sorptive extraction, *DMSPE* dispersive micro solid phase extraction

Depending on different extraction modes, extraction method can be divided into classic extraction methods and microextraction methods. Both classic extraction and microextraction methods are based on liquid phase extraction and solid phase extraction [62]. Classic extraction methods provide near-exhaustive extraction efficiency [45]. Compared with classic extraction methods, microextraction methods have the advantages of having high extraction efficiency and miniaturization of device and enrichment capacity making them more suitable for the analysis of plant hormone [47]. The extraction and clean-up techniques for trace compounds analysis include liquid–liquid extraction (LLE) [17, 21, 24, 30, 63–65], liquid phase microextraction (LPME) [52, 56, 66], dispersive liquid–liquid microextraction (DLLME) [40, 67], polymer monolith microextraction (PMME) [68], solid phase extraction (SPE) [26, 38, 55, 69–72], solid phase microextraction (SPME) [25, 34], magnetic solid phase extraction (MSPE) [16, 23, 73], matrix solid-phase dispersion (MSPD) [37], dispersive micro solid phase extraction (DMSPE) [74], ion pair stir bar sorptive extraction (SBSE) [1] and electromembrane extraction (EME) [75] (see Fig. 1).

The conventional LLE and SPE methods are widely used to analyze biological samples [76]. However, these sample pretreatment techniques are associated with drawbacks such as complicated operations, time-consuming, the requirement of relatively large amounts of sample and organic solvents, strong matrix effect (the interference and influence of matrix on analyte analysis) and the difficulty to automate. Flokova et al. [55] achieved rapid extraction of plant hormone with less sample consumption, less than 20 mg FW of *A. thaliana* leaf tissue in 10% methanol. They used single-step reversed-phase polymer-based SPE method which reduced the matrix effect and increased recovery of labile plant hormone.

Recent technological advancements have improved the miniaturization and automation of sample pretreatment methods. Compared with the traditional LLE and SPE methods, microextraction techniques such as DLLME, LPME, SPME and DMSPE applications in trace analysis are ever increasing. The solvent consumption, better extraction performance, minimized invasiveness and automated coupling with analytical instruments property of microextraction techniques have evidently benefited (Fig. 1) [62]. Cai et al. [77] developed a clean-up strategy employing a single-step dispersive solid-phase extraction (DSPE) combined with UPLC–MS/MS to obtain spatial–temporal information of 54 plant hormones including auxins, ABA, SA, JA, GAs and CKs. Wang et al. [75] used a combination of EME and LC–MS/MS to quantitatively detect six acidic plant hormones in 20 mg citrus leaf sample with a limit of detection (LOD) ranging from 0.1 to 10 ng mL^−1^. For more accuracy sample processing, sample pretreatment techniques require automation. Wang et al. [37] used an in-line coupled MSPD-MAX-MCX system to analyze a wide range of polar plant hormones in rice (200 mg). The proposed method showed higher extraction efficiency, lower matrix effect, ease of manipulation and time-saving characteristics. Recently, off-line procedures with highly efficient separation and detection sensitivity have been used to analyze plant hormone. However, the possible loss of trace plant hormone during additional process (compare with in-line procedures) has been an intractable challenge. Direct on-line analysis (coupling of sample preparation techniques directly to the separation and detection system) minimizes sample preparation steps and enables the effective pre-concentration and clean-up of plant samples [78]. Although these microextraction methods achieve effective plant hormone extraction, some of them are difficult to automate for on-line analysis.

### SPME strategies and advances

An ideal sample pretreatment technique for plant hormones analysis should be simple, rapid, selective, efficient, solvent-free, inexpensive, reproducible, highly accurate and avoids the degradation of analyses. For simultaneous quantitative analysis of multi-class plant hormones, the compatibility of an in vivo and automated sample pretreatment methods with dynamic and ultra-sensitive extraction capacity are required. SPME has advantages in these aspects [48, 50, 52, 79]. In SPME, sampling, extraction, concentration, and injection are integrated into a single step, which promotes miniaturization, automation, in vivo sampling and on-line analysis [80]. However, commercialized SPME sorbents exhibit lack selectivity which affects the distribution between analytes and the stationary phase. To address this challenge, several modified SPME sorbents have been designed to improve the sensitivity and selectivity of the extraction process [81]. Liu et al. [82] used a carbowax-coated fiber as SPME sorbent hyphenated with HPLC to detect auxin. They found that the modified sorbent had a relatively much higher extraction efficiency compared to polyacrylate fibers. Its dynamic range spanned over three orders of magnitude. The LOD/limit of quantification (LOQ) values of the target compounds in pure water were 0.149 (0.497), 0.442 (1.472), 0.121 (0.403) and 0.058 (0.193) μg L^−1^ for IAA, ABA, IBA and NAA, respectively. Song et al. [61] applied the SPME method incorporating on-cyclodextrin (–CD)-modified carbon nanotubes (CNTs) and a hollow fiber (HF) to analyze 1-naphthaleneacetic acid (NAA) and 2-naphthoxyacetic acid (2-NOA) in vegetables. This strategy allowed easy handling and provided environmentally friendly features as it minimized the amount of solvents. In most cases, plant hormones in plant tissues are determined simultaneously by combining SPME and HPLC [62, 76, 78]. Wang et al. [34] developed an automated and sensitive method to analyze the endogenous 28-epihomobrassinolide (28-epihomoBR) in *A. thaliana* by coupling on-line SPME with a polymer monolithic column and LC–MS (SPME-LC–MS). The poly (methacrylic acid-*co*-ethylene dimethacrylate) (poly (MAA-*co*-EDMA)) monolith was prepared in the capillary. The proposed SPME had satisfactory recovery (80.3–92.1%) and reproducibility (RSD 6.8–9.6%). Thus, SPME notably reduces assay duration and the difficulty of automation and improves in vivo analysis.

The application of SPME for in vivo analysis not only simplifies the extraction process, but also has the advantages of non-destructive extraction and easy to couple with mass spectrometry. Thus, this method facilitates on-site sampling, pretreatment and the detection of unstable analytes, making it suitable for long-term monitoring of signaling processes of plant hormones.

## Mass spectrometric analysis

Co-extraction of lipids and other interfering compounds is often eliminated by applying enrichment and purification methods. However simultaneous and accurate determination of individual or multi-class plant hormones in plant samples is still a great challenge due to the complex plant matrix [26, 80, 81, 83]. Various strategies including biological approaches (e.g. bioassays and immunoassays) physical and chemical analytical methods have been proposed to overcome the matrix effect of plant samples during the quantification of plant hormones [18]. Despite the high sensitivity of these analytical methods, most of them are not effective enough for the simultaneous analysis of multi-class plant hormones. MS-based technologies overcome these limitations. Successful exploitation of MS-based technologies for determining plant hormone from plant tissue satisfy the needs of high sensitivity. Furthermore, MS-based technologies can be combined with different chromatographic methods, which make it possible for high throughput analysis of plant samples [32, 55, 61, 75].

Plant hormone in plants were analyzed by GC–MS initially, in which the separation was dependent on using different partition coefficients between the gas phase and the stationary phase [20, 26, 56, 84–86]. However, most plant hormones are non-volatile, therefore a derivatization step is integral, not only it converts the non-volatile plant hormones to more volatile and thermally stable derivatives, but also for changing chromatographic behavior, improving detection sensitivity and selectivity of the analytes [87]. Based on the non-volatility property of most plant hormones, liquid chromatography has high separation performance when analyzing non-volatile compounds without the necessity of derivatization, which reduces possible loss of the target compound during derivatization procedure.

### LC–MS analysis

LC is by far the most common analytical technique used to qualitatively and quantitatively analyze trace plant hormone due to its high separation performance, high detection sensitivity, fast analytical speed and easy operation [60]. LC hyphenated with MS detection provides higher sensitivity and accuracy for the simultaneous analysis of multi-class plant hormone, which promotes research on the molecular biology of plant hormones [88]. Different LC–MS techniques used to analyze plant hormone are shown in Table 2.

**Table 2:** Overview of the mass spectrometry applications currently employed in plant hormone analysis

Analytes	Analytical technique	Plant matrix	Recovery (%)^a^	LODs	Linearity	References
JA, ABA, SA, BA, GAs	LC–ESI–MS/MS	Hamlin trees leave (20 mg FW)	34.6–50.3	0.03–3.00 ng mL^−1^	0.10–100.00 ng mL^−1^	[75]
JAs, SA, ABA, IAA	UHPLC–ESI–MS/MS	*A. thaliana* leaf (20 mg FW)	–	0.05–50 fmol	0.05–500.00 pmol	[55]
IAA, GAs, *t*Z,ABA	LC–ESI-IT-MS/MS	*A. thaliana* (100 mg FW)	70.0–100.0	0.55–170 fmol	5.00–1000.00 fmol	[63]
BRs	UHPLC-MS/MS	*Brassica napus* (50 mg FW)	30.9–88.9	1–50 pg	0.01–10.00 pmol	[20]
K, *i*P, BA, ABA, NAA	HPLC–MS/MS	Vermicompost (250 mg)	0.3–18.9	0.0015–0.3000 mg L^−1^	0.005–10.000 mg L^−1^	[14]
IAA, ABA, GAs, SA, JA, *t*Z, 6-BA, *i*P	LC–ESI–MS/MS	Oilseed rape	75.1–113.0	0.002–0.021 ng mL^−1^	0.0013–0.0210 ng mL^−1^	[89]
tZ, K, KR	UHPLC–ESI–MS/MS	Tobacco (100 mg FW)	68.8–103.0	2.4–47 pg mL^−1^	0.005–20.000 ng mL^−1^	[90]
ABA, IAA, JA, SA, GAs, *t*Z	UHPLC–ESI–MS/MS	*P. pinaster* Aiton. clones needles (200 ± 20 mg DW)	56.8–99.1	1.45–23.44 pg	0.040–2500.000 ng mL^−1^	[54]
BRs	UHPLC–ESI–MS/MS	*A. thaliana* (10 g FW)	76.9–86.1	2.00–8.00 ng L^−1^	10.00–10,000.00 ng L^−1^	[31]
GAs	UHPLC–ESI–MS/MS	Floral organs (about 80–250 μg)	64.0–107.0	Down to 5.41 amol	0.01–25 fmol	[21]
IAA, ABA, JA, SA, IBA, GAs	CE-ESI-TOF–MS	Rice leaves (3 g)	84.6–112.2	0.34–4.59 ng mL^−1^	1.30–850.00 ng mL^−1^	[91]
IAA, ABA, JA, SA, IBA, GAs	nano-LC–ESI-QTOF-MS	Rice leaves (5 mg FW)	88.3–104.3	1.05–122.4 pg mL^−1^	0.004–100.00 ng mL^−1^	[52]
ABA, IAA, IBA, GAs, SA	HPLC-ESI-QTOF-MS	Green seaweeds (100 mg FW)	80.0–92.0	0.5–1.0 μg mL^−1^	0.20–100.00 mg mL^−1^	[92]

^a^The recovery is relative recovery

The ion source allows large, non-volatile molecules to be analyzed directly from the liquid phase, which ionizes the neutral atom or molecule and fragment the ion beam generated from it [93]. Two of the frequently used ion sources in plant hormone analysis are electrospray ionization (ESI) and atmospheric pressure chemical ionization (APCI). ESI is preferred by most scientists as it has well balanced ionization efficiency for various classes of chemical compounds and is a soft ionization method [94]. It is, therefore, suitable for analysis of polar compounds because it does not require the rapid vaporization of solvents and ions can be generated in solution [95]. Since most plant hormones are polar in nature, ESI is comparatively a more suitable ion source for the characterization, identification and quantification of plant hormone in plant tissue [96].

#### Ionization efficiency of plant hormone analysis

The effectiveness of ionization method greatly influences the sensitivity of LC–MS analysis. Factors affecting the efficiency of ESI process includes composition and flow rate of mobile phase as well as matrix complexity of the plant sample [97]. The purification step of plant sample is crucial to improving the ionization efficiency, accuracy and sensitivity of LC–ESI–MS/MS analysis as it removes interfering co-eluting compounds which cause ion-suppression when using ESI, hence, affecting the ionization efficiency of plant hormone [98]. It is also important to optimize the composition, type and concentration of mobile phase and the purity of sample to ensure effective ionization during LC–ESI–MS analysis [99, 100]. Na^+^, K^+^ and NH_4_^+^ are commonly present in the solvent throughout the transportation or storage processes, which are classical additives and buffers of LC. Some mobile phase additives such as formic acid, acetic acid, trifluoroacetic acid are usually used to improve the ionization efficiency during MS detection. The chemical properties, concentration, and pH of the mobile phase additive have a significant effect on the LC–MS response [60, 87]. The type of appropriate additive added in mobile phase is critical to improve the chromatographic resolution, peak shape and ionization efficiency. Formic acid or acetic acid are the commonly used additives in the mobile phase to detect basic plant hormones in positive ion mode, whereas ammonium formate is used as the additive of the mobile phase to detect the acidic plant hormones in negative ion mode [101].

ESI is an ionization technique that converts ions in solution to gas-phase for MS analysis. The first step of ESI is the electrophoretic migration of anion and cation in the solution under an electric field to produce ions [102]. The efficiency of ion production depends on conductivity, which relies on the concentration of electrolyte. Suitable solvents for ESI include polar and moderately polar solvents and the most frequently used solvents are methanol, acetonitrile and water. Composition of the mobile phase for plant hormone analysis is usually water and acetonitrile (ACN) or methanol with suitable additives [103]. Methanol is an appropriate solvent for ESI–MS analysis of acidic compounds in the negative ionization mode. The response of some analytes in negative ESI–MS can be improved by applying methanol. In contrast, ACN is more suitable for analyzing in positive ion mode as it is an aprotic solvent [103]. The flow rate of mobile phase in the ESI ion source also influences the ionization process. Normally, smaller initial droplet is observed in the lower flow rate which decreases the number of fission cycles and the amount of solvents to be evaporated to form gas-phase ions, thus improving the electrospray effect [104].

Given the above-mentioned factors that affect the ionization efficiency, where the composition of the mobile phase affects the LC procedure. The LC analysis of plant hormone is often performed by combining water and methanol or acetonitrile with the addition of formic acid or ammonium formate as the mobile phase. The flow rate of mobile phase is often optimized at 0.2–0.5 mL min^−1^. Cai et al. [73] successfully separated and analyzed 8 CKs, IAA, ABA, JA and 10 GAs using a 27-min gradient of 0.05% FA H_2_O (A) and ACN (B) at a flow rate of 0.4 mL min^−1^. Suh et al. [75] characterized acidic plant hormones (JA, ABA, SA and GAs) in plant tissues using a linear gradient profile of 0.1% formic acid in water and (A) 0.1% formic acid in methanol (B) at 0.3 mL min^−1^.

#### Selection of mass analyzers for plant hormone analysis

**Fig. 2:**
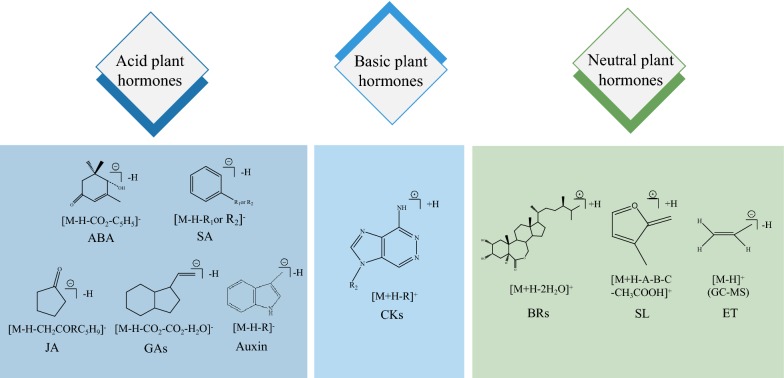
Fragmentation ions of multi-class plant hormones by triple quadrupole mass spectrometer (except ET)

Accurate identification of the mass-to-charge ratio of the ion peak is the principle of mass spectrometry and quantification. High resolution enhances the sensitivity of quantitative analysis [105]. One of the mainstream industries where LC–MS is constantly implemented is the doping control analysis, which employs different mass analyzers such as triple quadrupole (QqQ) [106], time of flight (TOF) [107] and quadrupole time of flight (QTOF) [108]. These qualitative and quantitative approaches are also applied in the detection of plant hormones. The use of multiple QqQ mass spectrometers to form a tandem mass spectrometry (MS/MS) not only enhances the qualitative capability of the mass spectrometer, but also retains the original quantitative capability of the quadrupole mass spectrometer. Several modes including full scan mode (Scan mode), selected ion monitor mode (SIM mode), multiple reaction monitor mode (MRM mode) and selected reaction monitor mode (SRM mode) are available in QqQ mass spectrometry [109]. Compared with the full scan mode in which all ions are simultaneously monitored, MRM mode provides higher selectivity and specificity to analyze multiple plant hormones. Fragmentation patterns of multi-class plant hormones in ESI–MS MRM mode (ET in Scan mode) are shown in Fig. 2. The conditions for MRM transition of plant hormones are listed in Table 3. Another mass analyzer widely used to detect plant hormones is TOF–MS [92]. TOF–MS exhibits faster detection speed, higher resolution and broader dynamic range of detection compared to QqQ-MS. The combination of TOF–MS and QqQ-MS provides broader application prospects (quadrupole time-of-flight mass spectrometer (QTOF-MS)) [98, 99, 110]. The quantitation of plant hormone by mass spectrometry is important for discovering the potential plant hormones in plants. Yang et al. [111] proposed identification of ripening degrees and cultivation regions of mulberries analysis, which employed high detection sensitivity and accuracy. Thus, LC–MS techniques provide an easy and robust method for plant hormone analysis.

**Table 3:** Optimized MRM parameters for plant hormone detection

Analytes	Scan mode	Precursor ion (Q1)	Collision energy (Q2)	Product ion (Q3)	References
*t*Z	+	220.2	26	136.1	[68]
NAA	+	185.1	− 10	158.1	[61]
6-BA	+	226.3	22	91.1	[54]
KT	+	216.0	22	148.0	[90]
IAA	−	174.0	− 14	129.6	[17]
IBA	−	203.0	− 16	158.3	[92]
IPA	−	188.0	− 16	58.9	[112]
ABA	−	263.1	− 12	153.9	[58]
JA	−	209.0	− 24	59.0	[25]
GA_3_	−	345.1	− 25	239.1	[113]
GA_4_	−	331.1	− 24	243.1	[75]
GA_7_	−	329.1	− 16	223.1	[63]
SA	−	137.1	− 25	92.8	[55]
BL	+	481.2	17	445.1	[20]
Sorgolactone	+	339.0	16	242	[114]

#### Chemical labeling

Over the past decades, the trend of plant hormone analysis has lied in a tiny amount sample consumption with sensitive performance [35, 115, 116]. However, most of the plant hormones were present in plant at ultra-low levels, while many classed of them exhibit with low ionization efficiency in MS [16, 40]. Benefit from high resolution of MS, chemical labeling may serve as a strong tool to enhance sensitivity and accuracy of determining plant hormones [11, 26], which overcome the problem by synthesizing series of stable isotope-coded reagents for chemical derivatization to the target compounds during sample pretreatment or MS detection procedure [117]. The stable isotope labeling affects the accuracy of the sample pretreatment procedure and apply the possibility to directly analyze plant hormones in the extract with relative reliability [118, 119]. When combine the light and heavy isotope labeled control and sample groups, the error that introduced during sample pretreatment step would compensate and the reliable relative quantification results then obtained [120]. Li et al. [112] were simultaneous relative quantification of 8 acidic plant hormones in sub-milligram amount of plant materials by stable isotope labeling by bromocholine bromide (BETA) and its deuterated counterpart D_9_-BETA, which improved sensitivity of 1–3 orders of magnitude. Furthermore, to improve the sensitivity of MS detections, amines are applied for the labelling (derivatization) procedure to form amides by making plant hormones easily ionized [31, 121]. Deng et al. [115] discovered a quaternary ammonium derivatization reagent (4-borono-*N*,*N*,*N*-trimethylbenzenaminium iodide) for ultrasensitive analysis of five BRs with vicinal diol functional groups on the side chain. The ionization efficiencies of the BRs enhanced by 1190–448,785 times and the minimal detectable amounts (MDA) of five target BRs were decreased to attomolar levels. The stable isotope labeling can improve the sensitivity and reproducibility of the plant hormone analytical procedures [19, 26, 112], which provide applicable capability in analyzing the plant hormone distribution and transportation in different plants.

## Mass spectrometric analysis of plant hormone

### Acidic plant hormones

#### Auxin, abscisic acid (ABA), jasmonic acid (JA) and salicylic acid (SA)

Typical acidic plant hormones comprise majority of the auxin, abscisic acid, jasmonic acid and salicylic acid [101, 102], which exhibit good response in MS negative ion mode. The quantitative analysis of these plant hormones in *A. thaliana*, wheat, *Aloe*, etc. have been realized by LC–MS/MS. Specific precursor-product ion pairs for each compound were monitored in MRM mode under the optimized fragmentor and collision energies [38, 55, 63]. A neutral CO_2_ molecule is easily lost during the ionization of plant hormone containing carboxyl group while [M-H-CO_2_]^−^ fragment ions are produced in the negative ion mode [54]. ABA only shows good ionization efficiency in the negative ion mode and releases [M-H]^−^ as a molecular ion peak [17]. JA liberates [M-H-COOH-C_5_H_9_]^−^ to form MW 58.7 product ion [55]. [M-H-R]^−^ fragment ions are produce from SA in negative ion mode [122] (Fig. 2). Specific precursor and product ions for each class of plant hormone were identified by distinctive fragmentation patterns to achieve accurately qualitative and quantitative analysis of multi-class plant hormones.

Xiao et al. [35] used a sensitive LC–MS method based on *N*-(acridin-9-yl)-2-bromoacetamide (AYBA) to examine the interactions between ABA, IAA and JA in germinated rice seeds. Good precisions with RSD 1.5–13.8% (intra-day) and 1.2–7.3% (inter-day) and acceptable recoveries (88.6–102.9%, n = 6) were achieved through this method. Luo et al. [16] developed a method for analyzing plant hormones including IAA, ABA, JA, GAs, CKs and BRs in 100 mg (FW) of *Brassica napus* L. The samples were purified and separated by sequential magnetic solid-phase extraction (MSPE) and then analyzed by an ultra-high performance liquid chromatography-tandem mass spectrometry (UHPLC–MS/MS) with the LOD (S/N = 3) ranging from 0.45 to 126.1 pg mL^−1^. The amount of plant tissues samples required has been reduced significantly from g (FW) to mg (FW). Cao et al. [70] developed a high-throughput method using liquid chromatography-triple quadrupole mass spectrometry (LC-QqQ-MS) for the profiling and quantification of 43 plant hormones and their major metabolites including auxins, ABA, JA, SA, CKs and GAs in a single sample extract. A binary solid-phase extraction with polymer anion and polymer cation exchange resins has been used as a sample pretreatment method to obtain LOD ranging from 0.03 to 29.7 fmol. The higher sensitivity of this method has enabled the detection of plant hormones at low concentration. Wu et al. [25] used in vivo SPME to detect GAs, JA and ABA plant hormones in 20 mg (FW) of aloe leaf with a spatial resolution of 3–8 mm^3^ and obtained an LOD of 60 pg g^−1^.

#### Gibberellin (GA)

Being a class of tetracyclic diterpenoid carboxylic acids with either ent-gibberellane or 20-nor-ent-gibberellane carbon skeletons, GAs comprise of at least 136 highly similar endogenous analogs [123]. GAs appear only at ultra-trace level in plants ranging from fmol to pmol per gram (FW). Apart from their poor detect-ability, the complexity and the similar chemical structures of ultra-trace GAs pose a greater analytical challenge [118]. LC-MS/MS is the most widely used method for simultaneous determination of multiple GAs, however their ESI–MS analysis has poor sensitivity because of low ionization efficiency in the negative ion mode [124]. Since tetracyclic diterpenoids are stable molecules, they are easily detected by mass spectrometry as a result of fragmentation through the cleavage between functional groups. MS/MS study of GAs is usually carried out in the negative ion mode because they do not form [M+H]^+^ in positive ion mode. Further, in the MS/MS analysis, the secondary fragmentation of GA_3_ in negative ion mode [M-H]^−^ produces [M-H-CO_2_]^−^ (m/z 301), [M-H-CO_2_-CO_2_]^−^ (m/z 257) and [M-H-CO_2_-CO_2_-H_2_O]^−^ (m/z 239) [125]. Under different collision energies, the high abundance of m/z 143 ion is observed in the MS^2^ of MS/MS spectrum indicating a stable product ion of GA_3_ [126]. Based on the fragmentation pathway, m/z 143 is generated by m/z 239 ions with the loss of [M-H-CO_2_-CO_2_-H_2_O-H_2_O-C_6_C_6_]^−^ (Fig. 2). Despite the clear fragmentation pattern of GAs, the detection of GAs is still challenging due to its extremely low content [21].

Chemical-label based LC–ESI–MS/MS is commonly used for analyze GAs by introducing positively charged moieties to improve the ionization efficiency and detection sensitivity. For example, derivatization reagents with quaternary and tertiary amine group were employed for GAs labeling to enhance the ionization efficiency (the derivatization methods are mentioned in Table 4) [121, 127]. These analytical methods are beneficial for elucidating the biosynthesis and distribution of GAs in plants.

**Table 4:** Derivatization methods and their validation parameters for plant hormone

Analytes	Matrix	Sample pretreatment	Derivatization conditions				Analytical technique	LOD	LOQ	Referencea
			Reagent (v/v ratios)	°C	min	Derivative structure				
BRs	Rice seeds (50 mg, FW)	In-tip SPE	4-PAMBA	–	–		LC–MS/MS	0.80–5.70 pg mL^−1^	2.70–19.00 pg mL^−1^	[119]
BRs	*A. thaliana* leaves (2 g, FW)	SPE	BPBA	− 20 °C	2 h		UHPLC-ESI-QqQ-MS	2.00–8.00 ng L^−1^	6.00–23.00 ng L^−1^	[31]
BRs	*A. thaliana* and rice (3 g, FW)	SPE	DMAPBA	40 °C	1 h		UPLC-QTOF MS	0.51–1.17 pg g^−1^	1.70–3.90 pg g^−1^	[128]
BRs	Rice leaves (5 ± 0.1 mg FW)	PT-SPE	BTBA	80 °C	30 min		UPLC-MS/MS	0.0065–0.021 ng g^−1^	0.022–0.068 ng g^−1^	[115]
BRs	Rice (250 mg, FW)	SPE	MPyBA	75 °C	–		UPLC-QTOF–MS	0.34–0.44 pg	1.12–1.45 pg	[129]
BRs	Tomato leaves (225 mg FW)	2DμSPE	m-APBA	–	–		HPLC–MS/MS	0.14–0.66 pg mL^−1^	0.47–2.20 pg mL^−1^	[57]
SA, IAA, IPA, IBA, ABA, JA, GA_4_	*A. thaliana* (0.8 mg DW)	SPE	BETA	95 °C	3 h		UPLC–MS/MS	0.61-1.85 pg mL^−1^	1.98-6.17 pg mL^−1^	[112]
SA, IAA,ABA, JA, GA_S_	Rice leaves (3 g, FW)	SPE	BTA/TEA = (1/1)	105 °C	10 min		CE-ESI-TOF-MS	0.34–4.59 ng mL^−1^	1.12–15.3 ng mL^−1^	[91]
GAs	*A. thaliana* leaves (0.3 mg, FW)	MSPD	BPTAB	85 °C	–		UPLC-MS/MS	0.16–1.31 pg mL^−1^	0.54–4.37 pg mL^−1^	[118]

The derivatization reaction is relatively difficult since it should be carried out in mild conditions because the structure of GAs undergoes different rearrangement reactions under varying pH. For example, GA_3_ and GA_7_ undergo lactone rearrangement under alkaline conditions (pH > 8.0) while a structural rearrangement of the C/D rings and hydration of the 16,17-double bond occurs in GA_1_ under strong acidic conditions (pH < 1.0). Bromocholinebromide (BETA) and its deuterated counterpart D_9_-BETA have been used to derivatize acidic plant hormones including GAs from sample extracts. ESI signal enhancements of 1–3 orders of magnitude were achieved under optimized conditions but a derivatization temperature of up to 95 °C was required [112]. 3-bromoactonyltrimethylammonium bromide (BTA) is used to increase the ionization efficiency of GAs. In a previous study, BTA was added prior to detection for nano-LC-ESI-Q-TOF–MS analyze GAs and LODs ranging from 1.05 to 122.4 pg mL^−1^ were obtained [130]. Li et al. [19] introduced a one-reagent labeling technique by using only *N*-(3-dimethylaminopropyl)-*N*-ethylcarbodiimide hydrochloride (EDC) to directly react with the ultra-trace GAs. This innovative labeling reaction was performed at pH 4.5 and 35 °C to meet the optimal conditions of pH 2.5–8.5 and a temperature of below 40 °C for analyze GAs. Protonated EDC reacts with the carboxyl group of GA to form an unstable O-acylurea, which subsequently rearranges to a stable and amine-irreplaceable N-acylurea product through O/N migration. The sensitivity is increased by 500- to 1000-fold as compared to unlabeled GAs, furthermore, this method can directly label GAs down to about 0.1 pM as it eliminates extra reagents.

### Basic plant hormones

#### Cytokinin (CK)

At present, the number of identified CKs exceeds 40, of which nucleosides (ribosides), nucleotides, and glycosides (O and N-glycosides) are free bases. The most naturally occurring CKs are N_6_-substituted adenine derivatives with an isoprenoid or aromatic side chain [106, 107]. The mass spectrometry studies of CKs is usually carried out in positive ion mode, since they are easily protonated ([M+H]^+^) [131]. After fragmentation, the loss of nucleosyl, glycosyl, or nucleotidyl group and the fragmentation of the N_6_-substituent for aglycons are observed in the spectrum. The representative plant hormone for CKs is *trans*-zeatin (*t*Z) [90]. When a precursor ion is formed, the N–H bond on the *t*Z side chain cleaves to form a m/z 136 product ion (C_5_H_4_N_5_) [132].

Antoniadi et al. [39] applied fluorescence-activated cell sorting of green fluorescent protein (GFP)-marked cell types, combined with in-tip micro-SPE and an ultra-high-sensitive MS technique to analyze the biosynthesis and homeostasis of CK at cellular level, which revealed a LOD of 0.01–1.0 fmol. Different LC systems can influence the sensitivity of MS. For instance, a sensitive assay of cytokinins was developed using PMME/HILIC/ESI–MS/MS which enhanced the MS sensitivity for cytokinins by threefold using HILIC as compared to the use of conventional reversed phase liquid chromatography (RPLC) with a mobile phase of 85% acetonitrile with 0.01% (v/v) formic acid and 15% water with 0.01% (v/v) formic acid. The LODs for the targets ranged from 0.0028 to 0.068 ng mL^−1^, and the intra-day and inter-day RSD of this method were less than 12.7% [68]. The choice of liquid chromatography system mainly depends on the enrichment ability of sample pretreatment and the sensitivity of mass spectrometry. Scott et al. [133] developed a conventional HPLC system that demonstrated good sensitivity without the need for an ultra-high performance liquid chromatography-mass spectrometry (UHPLC–MS) system. This method was used to screen 17 CKs (IAA, ABA and AMP), Ado and Ino within 15 min. The rapid analytical and high-throughput capabilities of this method enabled the analysis of close to 100 samples per day, with LOD ranging from 2 pM for (9G)Z to almost 750 pM for indole-3-acetic acid. This method is well suited for functional genomics platforms tailored for understanding CK metabolism.

### Neutral plant hormones

#### Brassinosteroid (BR)

All BRs contain a four-ring 5α-cholestan skeleton connected to a side chain. The diversity of BR structures is caused by variations in the A and B rings as well as the substituent groups of the side chains. BRs play a crucial role in many plant developmental processes although present in trace levels (down to 0.01–0.1 ng g^−1^ FW level) [92, 134–138]. Therefore, ideal sample pretreatment techniques should exhibit ultra-high sensitivity. Among the currently known BRs, brassinolide (BL) show the strongest activity, for BL fragmentation pattern, although the orientation of spatial position of the –OH in BL varies, ions generated from BL in the tandem mass spectrometry are substantially identical [139]. When BL fragmentation, as it shown in Fig. 2, the loss of water molecules and other neutral fragments are observed in the spectrum (BL lost two molecules of water to form the m/z 459 ion).

The commonly used detection methods such as MS are not suitable enough for BRs detection due to neutral nature with no ionizable group and the stronger hydrophobicity of BRs than other plant hormones [118, 124–126]. Therefore, the analysis of BRs requires highly selective sample preparation and highly sensitive detection with LC–MS after derivatization [29, 36, 40, 121, 126, 140]. Lv et al. [32] developed a novel hyphenated approach based on ultrasonic-assisted dispersive liquid–liquid microextraction (UA DLLME) after derivatization (using 9-phenanthreneboronic acid) for the determination of BL and obtained LOD of 8.0 ng L^−1^. Huo et al. [31] proposed a new labeling reagent, 2-bromopyridine-5-boronicacid (BPBA) for derivation brassinosteroids. This is a very simple and rapid labeling procedure that remarkably increases the sensitivity of BRs detection and LOD for the three BRs of 2.0 to 8.0 ng L^−1^. The automated extraction of BRs can be performed by an on-line SPME with polymer monolith coupled to LC–MS for the analysis of endogenous 28-epihomobrassinolide (28-epihomoBR) in *A. thaliana*. The BR was derivatized with 3-(trimethoxysilyl) propylmethacrylate prior to detection which revealed a highly sensitive result with LOD of 2.0 ng L^−1^ [34]. The combination of several purification methods provides an efficient and sensitive processing of plant samples. Recently, Wang et al. [37] quantified six endogenous BRs with good compatibility and sensitivity by eliminating non-polar, polar and ionizable interferences using a matrix in-line MSPD–MAX–MCX coupled with HPLC–MS/MS to obtain LODs ranging from 0.008 to 0.04 ng mL^−1^. In another study, in situ derivatization (ISD) was introduced and coupled with multiple sample preparation methods to simplify the process of extraction and derivatization [141]. Luo et al. [42] developed a solid phase boronate affinity labeling (SPBAL) and extraction technique, followed by a desorption & salt-induced phase transition extraction (SPTE) for further purification to rapidly determine endogenous BRs in plant tissues. The addition of a boronate affinity labeling reagent (4-PAMBA) for ISD lead to a 923–15,000-fold increase in sensitivity of BRs detection, with LODs ranging between 1.4 and 2.8 pg mL^−1^.

#### Strigolactone (SL)

Strigolactones (SLs) have been suggested to act as a long-distance branching factor that suppresses the growth of preformed axillary shoot buds. They are derived from carotenoids and were recently recognized as a new family of plant hormone [59, 91, 111, 142]. Typically, the natural SLs identified so far consist of tetracyclic skeleton (A, B, C, and D rings) with a tricyclic lactone (ABC rings) connected by an enol ether group to an α,β-unsaturated furanone moiety (D ring) [114]. SLs show relatively lower ionization efficiency because of their neutral structure which is similar to BRs which cause interference during MS detection. The lactone structure of C and D rings in SL molecules exhibits lower stability as they can hydrolyze in acidic or alkaline conditions leading to a more complicated sample preparation and instrumental analysis [44]. Except for the same structure of D ring, some of the ABC rings in SLs contain –OH, –CH_3_, –COOH, –CH_2_CO and –O– groups. The compounds with these functional groups including D ring can easily be cleaved to form fragment ions during the cleavage process in MS detection. SLs are usually detected in the positive ion mode. A typical SLs-GR24 (C_17_H_14_O_5_) has a molecular weight of 298, and the main fragment ions formed are m/z = 97 and m/z = 185 [44, 114, 143, 144]. The typical product ion of SLs (m/z = 97) is mainly the enol ether found between the ABC ring and the D ring in the GR_24_ structure [144].

Yoneyama et al. [114] analyzed SLs in root exudates from 12 *Fabaceae* plants employing LC–MS/MS to clarify the regulation of SL production and revealed that exudation is closely related to the nutrient acquisition strategy of plants. Xie et al. [44] also used LC–MS/MS to characterize five different stimulants including four SLs from the root exudates of tobacco providing a valuable analytical strategy for determination of SLs. Foo et al. [143] used UPLC-MS/MS to quantify SL levels in the root exudate of 30-day-old rms1-2T and wild-type plants using deuterium-labeled internal standards. This was the first direct evidence that shooting does not majorly contribute to the SL levels in roots. Kohlen et al. [144] performed SL detection using HPLC -MS/MS, and the MRM transitions of [M+H/Na]^+^ > [M+H/Na-D ring]^+^ and [M+H/Na]^+^ > [D ring]^+^ were selected. Two SLs (orobanchol and orobanchyl acetate) were identified in *Arabidopsis* and the presence of a third (5-deoxystrigol) SL was discovered. These results show that xylem-transported SLs contribute to the regulation of shoot architectural response to phosphate-limiting conditions.

## Conclusions

Major technological advancements in sample pretreatment methods and mass spectrometric methods in recent years has substantially facilitated plant hormone analysis [117, 145–148]. To date, MS studies have identified many plant hormones and their metabolites that might be involved in the molecular mechanisms and physiological functions of plant development, but the analysis at unicellular level has remained a challenge. Several large-scale studies on plant hormone have been reported, these studies have promoted our understanding on the dynamic spatial–temporal distribution of plant hormone [19, 35, 77]. Current sample pretreatment methods for plant hormone is expected to be further optimized for realizing the in situ, real-time and high spatial resolution. The frontier research of analytical methodology will broaden the development of sample pretreatment techniques and derivatization strategies for more accurate characterization of plant hormone.

Going forward, the existing methods need to be improved to enhance the efficiency of sample treatment and chromatographic-mass spectrometry analysis of plant hormone in the following aspects:

1. Sample pretreatment methods with the ability to achieve real-time in situ and in vivo analysis of the spatial–temporal distribution of plant hormone are required to facilitate further studies on plant hormone regarding the synthesis, metabolism, transportation pathway and functional effects. Therefore, it is anticipated that the development of highly efficient sample pretreatment methods for in vivo and real-time analysis. The efficiency methods are likely to dominate future research to improve the characterization of the distribution of multi-class plant hormones within a whole plant or specific plant organs in single cell orientation.
2. Several methods have been developed for multi-class plant hormones analysis, but even the most influential methods are expected to improve the selectivity prior to analyzing different plant hormones isomers belonging to the same class with structural similarities. Recent advancements in the analytical methods with micro-separation ability, excellent selectivity and high sensitivity features would facilitate more comprehensive real-time monitoring of plant hormone during growth and development.
3. Mass spectrometric analysis is highly suitable for plant hormone analysis and has witnessed widespread application in recent years. Further studies would anticipate to develop derivatization methods for ultra-trace compounds analysis, as derivatization methods improve the analytical sensitivity and the spatial–temporal resolution of plant hormone. Furthermore, the establishment of a mass spectral database for plant hormone is crucial for accurate qualitative and quantitative analysis. Lastly, such a database may act as a tool for advancing scientific theories and for the potential discovery of new plant hormones.

## Data Availability

Not applicable.

## Abbreviations

CK: Cytokinin
ABA: Abscisic acid
GA: Gibberellin
BR: Brassinosteroid
SA: Salicylic acid
JA: Jasmonic acid
ET: Ethylene
SL: Strigolactone
FW: Fresh weight
MS: Mass spectrometry
GC-MS: Gas chromatography–mass spectrometry
LC-MS: Liquid chromatography-mass spectrometry
IT: Ion trap
Q-TOF: Quadrupole time-of-flight
QQQ: Triple quadrupole instruments
LLE: Liquid–liquid extraction
LPME: Liquid phase microextraction
DLLME: Dispersive liquid–liquid microextraction
PMME: Polymer monolith microextraction
SPE: Solid phase extraction
SPME: Solid phase microextraction
MSPE: Magnetic solid phase extraction
SBSE: Ion pair stir bar sorptive extraction
EME: Electromembrane extraction
CE: Capillary electrophoresis
DSPE: Dispersive solid-phase extraction
HPLC: High performance liquid chromatography
UHPLC: Ultra high performance liquid chromatography-mass spectrometry
RPLC: Reversed phase liquid chromatography
LOD: Limit of detection
LOQ: Limit of quantitation
–CD: On-cyclodextrin
NAA: Naphthaleneacetic acid
2-NOA: 2-Naphthoxyacetic acid
tZ: Trans-zeatin
28-epihomoBR: 28-Epihomobrassinolide
poly(MAA-co-EDMA): Poly(methacrylic acid-co-ethylene dimethacrylate)
RSD: Relative standard deviation
ESI: Electrospray ionization
APCI: Atmospheric pressure chemical ionization
ACN: Acetonitrile
QqQ: Triple quadrupole
TOF: Time of flight
QTOF: Quadrupole time of flight
MS/MS: Tandem mass spectrometry
Scan mode: Scan mode
SIM mode: Selected ion monitor mode
MRM mode: Multiple reaction monitor mode
SRM mode: Selected reaction monitor mode
ESI-MS: Electrospray ionization mass spectrometry
QTOF-MS: Quadrupole time-of-flight mass spectrometer
LC-QqQ-MS: Liquid chromatography-triple quadrupole mass spectrometry
–COOH: Carboxyl groups
–OH: Hydroxyl groups
AYBA: *N*-(acridin-9-yl)-2-bromoacetamide
BETA: Bromocholinebromide
BTA: 3-Bromoactonyltrimethylammonium bromide
EDC: *N*-(3-dimethylaminopropyl)-*N*-ethylcarbodiimide hydrochloride
SPBAL: Solid phase boronate affinity labeling
SPTE: Salt-induced phase transition extraction

## References

[1] Wang W, He M, Chen B, Hu B (2017). Simultaneous determination of acidic phytohormones in cucumbers and green bean sprouts by ion-pair stir bar sorptive extraction-high performance liquid chromatography. Talanta.

[2] Miransari M, Smith DL (2014). Plant hormones and seed germination. Environ Exp Bot.

[3] Peleg Z, Blumwald E (2011). Hormone balance and abiotic stress tolerance in crop plants. Curr Opin Plant Biol.

[4] Santner A, Estelle M (2009). Recent advances and emerging trends in plant hormone signalling. Nature.

[5] Sánchez-Rodríguez C, Rubio-Somoza I, Sibout R, Persson S (2010). Phytohormones and the cell wall in *Arabidopsis* during seedling growth. Trends Plant Sci.

[6] Li H-H, Hao R-L, Wu S-S, Guo P-C, Chen C-J, Pan L-P (2011). Occurrence, function and potential medicinal applications of the phytohormone abscisic acid in animals and humans. Biochem Pharmacol.

[7] Osterc G, Štampar F (2011). Differences in endo/exogenous auxin profile in cuttings of different physiological ages. J Plant Physiol.

[8] Wei K, Wang L, Cheng H, Zhang C, Ma C, Zhang L (2013). Identification of genes involved in indole-3-butyric acid-induced adventitious root formation in nodal cuttings of *Camellia sinensis* (L.) by suppression subtractive hybridization. Gene..

[9] Bahyrycz A, Konopińska D (2007). Plant signalling peptides: some recent developments. J Pept Sci.

[10] Porfírio S, Gomes da Silva MDR, Peixe A, Cabrita MJ, Azadi P (2016). Current analytical methods for plant auxin quantification—a review. Anal Chim Acta..

[11] Böhmer M, Schroeder JI (2011). Quantitative transcriptomic analysis of abscisic acid-induced and reactive oxygen species-dependent expression changes and proteomic profiling in *Arabidopsis* suspension cells: ABA- and ROS-regulated expression changes. Plant J..

[12] Bowman JL, Briginshaw LN, Fisher TJ, Flores-Sandoval E (2019). Something ancient and something neofunctionalized—evolution of land plant hormone signaling pathways. Curr Opin Plant Biol.

[13] Eggert K, von Wirén N (2017). Response of the plant hormone network to boron deficiency. New Phytol.

[14] Samodelov SL, Zurbriggen MD (2017). Quantitatively understanding plant signaling: novel theoretical-experimental approaches. Trends Plant Sci.

[15] Tarkowská D, Novák O, Floková K, Tarkowski P, Turečková V, Grúz J (2014). Quo vadis plant hormone analysis?. Planta.

[16] Luo X-T, Cai B-D, Chen X, Feng Y-Q (2017). Improved methodology for analysis of multiple phytohormones using sequential magnetic solid-phase extraction coupled with liquid chromatography-tandem mass spectrometry. Anal Chim Acta.

[17] Pan X, Welti R, Wang X (2010). Quantitative analysis of major plant hormones in crude plant extracts by high-performance liquid chromatography–mass spectrometry. Nat Protoc.

[18] Du F, Ruan G, Liu H (2012). Analytical methods for tracing plant hormones. Anal Bioanal Chem.

[19] Li D, Guo Z, Chen Y (2016). Direct derivatization and quantitation of ultra-trace gibberellins in sub-milligram fresh plant organs. Mol Plant..

[20] Oklestkova J, Tarkowská D, Eyer L, Elbert T, Marek A, Smržová Z (2017). Immunoaffinity chromatography combined with tandem mass spectrometry: a new tool for the selective capture and analysis of brassinosteroid plant hormones. Talanta.

[21] Li D, Guo Z, Liu C, Li J, Xu W, Chen Y (2017). Quantification of near-attomole gibberellins in floral organs dissected from a single *Arabidopsis thaliana* flower. Plant J..

[22] Manzi M, Gómez-Cadenas A, Arbona V (2015). Rapid and reproducible determination of active gibberellins in citrus tissues by UPLC/ESI-MS/MS. Plant Physiol Biochem.

[23] Chang Y-H, Huang C-W, Fu S-F, Wu M-Y, Wu T, Lin Y-W (2017). Determination of salicylic acid using a magnetic iron oxide nanoparticle-based solid-phase extraction procedure followed by an online concentration technique through micellar electrokinetic capillary chromatography. J Chromatogr A.

[24] Rawlinson C, Kamphuis LG, Gummer JPA, Singh KB, Trengove RD (2015). A rapid method for profiling of volatile and semi-volatile phytohormones using methyl chloroformate derivatisation and GC–MS. Metabolomics.

[25] Wu Q, Wu D, Guan Y (2014). Polyaniline sheathed electrospun nanofiber bar for in vivo extraction of trace acidic phytohormones in plant tissue. J Chromatogr A.

[26] Tarkowská D, Novák O, Oklestkova J, Strnad M (2016). The determination of 22 natural brassinosteroids in a minute sample of plant tissue by UHPLC–ESI–MS/MS. Anal Bioanal Chem.

[27] Vallarino JG, Osorio S (2016). Simultaneous Determination of plant hormones by GC-TOF-MS. Methods Mol Biol.

[28] Chen H, Guo X-F, Zhang H-S, Wang H (2011). Simultaneous determination of phytohormones containing carboxyl in crude extracts of fruit samples based on chemical derivatization by capillary electrophoresis with laser-induced fluorescence detection. J Chromatogr B.

[29] Verslues PE (2017). Rapid quantification of abscisic acid by GC-MS/MS for studies of abiotic stress response. Plant stress tolerance: methods and protocols. Methods Mol Biol.

[30] Nehela Y, Hijaz F, Elzaawely AA, El-Zahaby HM, Killiny N (2016). Phytohormone profiling of the sweet orange (*Citrus sinensis* (L.) Osbeck) leaves and roots using GC–MS-based method. J Plant Physiol..

[31] Huo F, Wang X, Han Y, Bai Y, Zhang W, Yuan H (2012). A new derivatization approach for the rapid and sensitive analysis of brassinosteroids by using ultra high performance liquid chromatography-electrospray ionization triple quadrupole mass spectrometry. Talanta.

[32] Lv T, Zhao X-E, Zhu S, Ji Z, Chen G, Sun Z (2014). Development of an efficient HPLC fluorescence detection method for brassinolide by ultrasonic-assisted dispersive liquid-liquid microextraction coupled with derivatization. Chromatographia.

[33] Ziegler J, Qwegwer J, Schubert M, Erickson JL, Schattat M, Bürstenbinder K (2014). Simultaneous analysis of apolar phytohormones and 1-aminocyclopropan-1-carboxylic acid by high performance liquid chromatography/electrospray negative ion tandem mass spectrometry via 9-fluorenylmethoxycarbonyl chloride derivatization. J Chromatogr A.

[34] Wang X, Ma Q, Li M, Chang C, Bai Y, Feng Y (2013). Automated and sensitive analysis of 28-epihomobrassinolide in *Arabidopsis thaliana* by on-line polymer monolith microextraction coupled to liquid chromatography–mass spectrometry. J Chromatogr A.

[35] Xiao H-M, Cai W-J, Ye T-T, Ding J, Feng Y-Q (2018). Spatio-temporal profiling of abscisic acid, indoleacetic acid and jasmonic acid in single rice seed during seed germination. Anal Chim Acta.

[36] Sun L-J, Feng Q-M, Yan Y-F, Pan Z-Q, Li X-H, Song F-M (2014). Paper-based electroanalytical devices for in situ determination of salicylic acid in living tomato leaves. Biosens Bioelectron.

[37] Wang L, Duan C, Wu D, Guan Y (2014). Quantification of endogenous brassinosteroids in sub-gram plant tissues by in-line matrix solid-phase dispersion–tandem solid phase extraction coupled with high performance liquid chromatography–tandem mass spectrometry. J Chromatogr A.

[38] Nordström A, Tarkowski P, Tarkowska D, Dolezal K, Åstot C, Sandberg G (2004). Derivatization for LC-electrospray ionization-MS: a tool for improving reversed-phase separation and ESI responses of bases, ribosides, and intact nucleotides. Anal Chem.

[39] Antoniadi I, Plačková L, Simonovik B, Doležal K, Turnbull C, Ljung K (2015). Cell-type-specific cytokinin distribution within the arabidopsis primary root apex. Plant Cell..

[40] Lu Q, Zhang W, Gao J, Lu M, Zhang L, Li J (2015). Simultaneous determination of plant hormones in peach based on dispersive liquid–liquid microextraction coupled with liquid chromatography–ion trap mass spectrometry. J Chromatogr B.

[41] Okazaki Y, Kamide Y, Hirai MY, Saito K (2013). Plant lipidomics based on hydrophilic interaction chromatography coupled to ion trap time-of-flight mass spectrometry. Metabolomics.

[42] Luo X-T, Cai B-D, Yu L, Ding J, Feng Y-Q (2018). Sensitive determination of brassinosteroids by solid phase boronate affinity labeling coupled with liquid chromatography-tandem mass spectrometry. J Chromatogr A.

[43] Pan X, Wang X (2009). Profiling of plant hormones by mass spectrometry. J Chromatogr B.

[44] Xie X, Kusumoto D, Takeuchi Y, Yoneyama K, Yamada Y, Yoneyama K (2007). 2′-Epi-orobanchol and solanacol, two unique strigolactones, germination stimulants for root parasitic weeds, produced by tobacco. J Agric Food Chem.

[45] Sajid M, Płotka-Wasylka J (2018). Combined extraction and microextraction techniques: recent trends and future perspectives. TrAC Trends Anal Chem.

[46] Abedi G, Talebpour Z, Jamechenarboo F (2018). The survey of analytical methods for sample preparation and analysis of fragrances in cosmetics and personal care products. TrAC Trends Anal Chem.

[47] Kataoka H, Saito K (2011). Recent advances in SPME techniques in biomedical analysis. J Pharm Biomed Anal.

[48] Souza Silva EA, Risticevic S, Pawliszyn J (2013). Recent trends in SPME concerning sorbent materials, configurations and in vivo applications. TrAC Trends Anal Chem.

[49] Song X-Y, Chen J, Shi Y-P (2017). Different configurations of carbon nanotubes reinforced solid-phase microextraction techniques and their applications in the environmental analysis. TrAC Trends Anal Chem.

[50] Xu J, Chen G, Huang S, Qiu J, Jiang R, Zhu F (2016). Application of in vivo solid-phase microextraction in environmental analysis. TrAC Trends Anal Chem.

[51] Du F, Bai Y, Bai Y, Liu H (2010). Quantitative detection of trace systemins in *Solanaceous* plants by immunoaffinity purification combined with liquid chromatography/electrospray quadrupole time-of-flight mass spectrometry. Anal Chem.

[52] Xu C-H, Chen G-S, Xiong Z-H, Fan Y-X, Wang X-C, Liu Y (2016). Applications of solid-phase microextraction in food analysis. TrAC Trends Anal Chem.

[53] Bai Y, Du F, Bai Y, Liu H (2010). Determination strategies of phytohormones: recent advances. Anal Methods.

[54] Delatorre C, Rodríguez A, Rodríguez L, Majada JP, Ordás RJ, Feito I (2017). Hormonal profiling: development of a simple method to extract and quantify phytohormones in complex matrices by UHPLC–MS/MS. J Chromatogr B.

[55] Floková K, Tarkowská D, Miersch O, Strnad M, Wasternack C, Novák O (2014). UHPLC–MS/MS based target profiling of stress-induced phytohormones. Phytochemistry.

[56] Zhang X, Jiang X, Hao Z, Qu K (2019). Advances in online methods for monitoring microbial growth. Biosens Bioelectron.

[57] Wu Q, Wu D, Shen Z, Duan C, Guan Y (2013). Quantification of endogenous brassinosteroids in plant by on-line two-dimensional microscale solid phase extraction-on column derivatization coupled with high performance liquid chromatography–tandem mass spectrometry. J Chromatogr A.

[58] Bai Y, Zhang J, Bai Y, Liu H (2012). Direct analysis in real time mass spectrometry combined with single-drop liquid–liquid–liquid microextraction for the rapid analysis of multiple phytohormones in fruit juice. Anal Bioanal Chem.

[59] Dong SY, Yang Z, Zhang PH, Hu Q, Huang TL (2012). Comparative study of hollow-fiber liquid-phase micro-extraction and an aqueous two-phase system for determination of phytohormones in soil. Anal Bioanal Chem.

[60] Ding J, Mao L-J, Yuan B-F, Feng Y-Q (2013). A selective pretreatment method for determination of endogenous active brassinosteroids in plant tissues: double layered solid phase extraction combined with boronate affinity polymer monolith microextraction. Plant Methods..

[61] Song X-Y, Ha W, Chen J, Shi Y-P (2014). Application of β-cyclodextrin-modified, carbon nanotube-reinforced hollow fiber to solid-phase microextraction of plant hormones. J Chromatogr A.

[62] Werner J, Grześkowiak T, Zgoła-Grześkowiak A, Stanisz E (2018). Recent trends in microextraction techniques used in determination of arsenic species. TrAC Trends Anal Chem.

[63] Izumi Y, Okazawa A, Bamba T, Kobayashi A, Fukusaki E (2009). Development of a method for comprehensive and quantitative analysis of plant hormones by highly sensitive nanoflow liquid chromatography–electrospray ionization-ion trap mass spectrometry. Anal Chim Acta.

[64] Li G, Liu S, Sun Z, Xia L, Chen G, You J (2015). A simple and sensitive HPLC method based on pre-column fluorescence labelling for multiple classes of plant growth regulator determination in food samples. Food Chem.

[65] Müller A, Düchting P, Weiler E (2002). A multiplex GC-MS/MS technique for the sensitive and quantitative single-run analysis of acidic phytohormones and related compounds, and its application to *Arabidopsis thaliana*. Planta.

[66] Asadi M, Dadfarnia S, Haji Shabani AM, Abbasi B (2016). Hollow fiber liquid phase microextraction method combined with high-performance liquid chromatography for simultaneous separation and determination of ultra-trace amounts of naproxen and nabumetone in cow milk, water, and biological samples. Food Anal Methods.

[67] Porfírio S, Sonon R, Gomes da Silva MDR, Peixe A, Cabrita MJ, Azadi P (2016). Quantification of free auxins in semi-hardwood plant cuttings and microshoots by dispersive liquid–liquid microextraction/microwave derivatization and GC/MS analysis. Anal Methods..

[68] Liu Z, Wei F, Feng Y-Q (2010). Determination of cytokinins in plant samples by polymer monolith microextraction coupled with hydrophilic interaction chromatography-tandem mass spectrometry. Anal Methods.

[69] Dobrev PI, Havlíček L, Vágner M, Malbeck J, Kamínek M (2005). Purification and determination of plant hormones auxin and abscisic acid using solid phase extraction and two-dimensional high performance liquid chromatography. J Chromatogr A.

[70] Cao Z-Y, Sun L-H, Mou R-X, Zhang L-P, Lin X-Y, Zhu Z-W (2016). Profiling of phytohormones and their major metabolites in rice using binary solid-phase extraction and liquid chromatography-triple quadrupole mass spectrometry. J Chromatogr A.

[71] Ma Z, Ge L, Lee ASY, Yong JWH, Tan SN, Ong ES (2008). Simultaneous analysis of different classes of phytohormones in coconut (*Cocos nucifera* L.) water using high-performance liquid chromatography and liquid chromatography–tandem mass spectrometry after solid-phase extraction. Anal Chim Acta..

[72] Pan J, Huang Y, Liu L, Hu Y, Li G (2013). A novel fractionized sampling and stacking strategy for online hyphenation of solid-phase-based extraction to ultra-high performance liquid chromatography for ultrasensitive analysis. J Chromatogr A.

[73] Cai B-D, Yin J, Hao Y-H, Li Y-N, Yuan B-F, Feng Y-Q (2015). Profiling of phytohormones in rice under elevated cadmium concentration levels by magnetic solid-phase extraction coupled with liquid chromatography tandem mass spectrometry. J Chromatogr A.

[74] Yanping X, Yuluan W, Hao D (2017). Dispersive micro solid phase extraction (DMSPE) using polymer anion exchange (PAX) as the sorbent followed by UPLC-MS/MS for the rapid determination of four bisphenols in commercial edible oils. J Chromatogr A..

[75] Suh JH, Han SB, Wang Y (2018). Development of an improved sample preparation platform for acidic endogenous hormones in plant tissues using electromembrane extraction. J Chromatogr A.

[76] Niu Z, Zhang W, Yu C, Zhang J, Wen Y (2018). Recent advances in biological sample preparation methods coupled with chromatography, spectrometry and electrochemistry analysis techniques. TrAC Trends Anal Chem.

[77] Cai W-J, Ye T-T, Wang Q, Cai B-D, Feng Y-Q (2016). A rapid approach to investigate spatiotemporal distribution of phytohormones in rice. Plant Methods..

[78] Valsecchi S, Polesello S, Mazzoni M, Rusconi M, Petrovic M (2015). On-line sample extraction and purification for the LC–MS determination of emerging contaminants in environmental samples. Trends Environ Anal Chem..

[79] Fernández-Amado M, Prieto-Blanco MC, López-Mahía P, Muniategui-Lorenzo S, Prada-Rodríguez D (2016). Strengths and weaknesses of in-tube solid-phase microextraction: a scoping review. Anal Chim Acta.

[80] Reyes-Garcés N, Gionfriddo E (2019). Recent developments and applications of solid phase microextraction as a sample preparation approach for mass-spectrometry-based metabolomics and lipidomics. TrAC Trends Anal Chem.

[81] Lashgari M, Yamini Y (2019). An overview of the most common lab-made coating materials in solid phase microextraction. Talanta.

[82] Liu H-T, Li Y-F, Luan T-G, Lan C-Y, Shu W-S (2007). Simultaneous determination of phytohormones in plant extracts using SPME and HPLC. Chromatographia.

[83] Xu J, Zheng J, Tian J, Zhu F, Zeng F, Su C (2013). New materials in solid-phase microextraction. TrAC Trends Anal Chem.

[84] Queiroz MEC, Melo LP (2014). Selective capillary coating materials for in-tube solid-phase microextraction coupled to liquid chromatography to determine drugs and biomarkers in biological samples: a review. Anal Chim Acta.

[85] Nawała J, Dawidziuk B, Dziedzic D, Gordon D, Popiel S (2018). Applications of ionic liquids in analytical chemistry with a particular emphasis on their use in solid-phase microextraction. TrAC Trends Anal Chem.

[86] Wei X, Shi B, Koo I, Yin X, Lorkiewicz P, Suhail H (2017). Analysis of stable isotope assisted metabolomics data acquired by GC-MS. Anal Chim Acta.

[87] La Nasa J, Modugno F, Aloisi M, Lluveras-Tenorio A, Bonaduce I (2018). Development of a GC/MS method for the qualitative and quantitative analysis of mixtures of free fatty acids and metal soaps in paint samples. Anal Chim Acta.

[88] Steinmann D, Ganzera M (2011). Recent advances on HPLC/MS in medicinal plant analysis. J Pharm Biomed Anal.

[89] Górka B, Wieczorek PP (2017). Simultaneous determination of nine phytohormones in seaweed and algae extracts by HPLC-PDA. J Chromatogr B.

[90] Du F, Sun L, Zhen X, Nie H, Zheng Y, Ruan G (2015). High-internal-phase-emulsion polymeric monolith coupled with liquid chromatography–electrospray tandem mass spectrometry for enrichment and sensitive detection of trace cytokinins in plant samples. Anal Bioanal Chem.

[91] Chen M-L, Huang Y-Q, Liu J-Q, Yuan B-F, Feng Y-Q (2011). Highly sensitive profiling assay of acidic plant hormones using a novel mass probe by capillary electrophoresis-time of flight-mass spectrometry. J Chromatogr B.

[92] Gupta V, Kumar M, Brahmbhatt H, Reddy CRK, Seth A, Jha B (2011). Simultaneous determination of different endogenetic plant growth regulators in common green seaweeds using dispersive liquid–liquid microextraction method. Plant Physiol Biochem.

[93] Cui K, Lin Y, Zhou X, Li S, Liu H, Zeng F (2015). Comparison of sample pretreatment methods for the determination of multiple phytohormones in plant samples by liquid chromatography–electrospray ionization-tandem mass spectrometry. Microchem J.

[94] Kumar BR (2017). Application of HPLC and ESI-MS techniques in the analysis of phenolic acids and flavonoids from green leafy vegetables (GLVs). J Pharm Anal..

[95] Forcisi S, Moritz F, Kanawati B, Tziotis D, Lehmann R, Schmitt-Kopplin P (2013). Liquid chromatography–mass spectrometry in metabolomics research: mass analyzers in ultra high pressure liquid chromatography coupling. J Chromatogr A.

[96] Vikse KL, Ahmadi Z, Scott McIndoe J (2014). The application of electrospray ionization mass spectrometry to homogeneous catalysis. Coord Chem Rev.

[97] Breitbach ZS, Wanigasekara E, Dodbiba E, Schug KA, Armstrong DW (2010). Mechanisms of ESI-MS selectivity and sensitivity enhancements when detecting anions in the positive mode using cationic pairing agents. Anal Chem.

[98] Ghosh C, Shinde CP, Chakraborty BS (2012). Influence of ionization source design on matrix effects during LC–ESI-MS/MS analysis. J Chromatogr B.

[99] Rainville PD, Smith NW, Cowan D, Plumb RS (2012). Comprehensive investigation of the influence of acidic, basic, and organic mobile phase compositions on bioanalytical assay sensitivity in positive ESI mode LC/MS/MS. J Pharm Biomed Anal.

[100] Kruve A, Kaupmees K (2017). Predicting ESI/MS signal change for anions in different solvents. Anal Chem.

[101] Yeniceli D, Şener E, Korkmaz OT, Doğrukol-Ak D, Tuncel N (2011). A simple and sensitive LC–ESI-MS (ion trap) method for the determination of bupropion and its major metabolite, hydroxybupropion in rat plasma and brain microdialysates. Talanta.

[102] Higashi T, Ogawa S (2016). Chemical derivatization for enhancing sensitivity during LC/ESI–MS/MS quantification of steroids in biological samples: a review. J Steroid Biochem Mol Biol.

[103] Huffman BA, Poltash ML, Hughey CA (2012). Effect of polar protic and polar aprotic solvents on negative-ion electrospray ionization and chromatographic separation of small acidic molecules. Anal Chem.

[104] Luo Q, Page JS, Tang K, Smith RD (2007). MicroSPE-nanoLC-ESI-MS/MS using 10-μm-i.d. silica-based monolithic columns for proteomics. Anal Chem..

[105] Goswami D, Zhang J, Bondarenko PV, Zhang Z (2018). MS-based conformation analysis of recombinant proteins in design, optimization and development of biopharmaceuticals. Methods.

[106] Bischoff R, Bronsema KJ, van de Merbel NC, Bronsema KJ, van de Merbel NC (2013). Analysis of biopharmaceutical proteins in biological matrices by LC-MS/MS I. Sample preparation. TrAC Trends Anal Chem..

[107] Lasch P, Jacob D, Grunow R, Schwecke T, Doellinger J (2016). Matrix-assisted laser desorption/ionization time-of-flight (MALDI-TOF) mass spectrometry (MS) for the identification of highly pathogenic bacteria. TrAC Trends Anal Chem.

[108] Drotleff B, Hallschmid M, Lämmerhofer M (2018). Quantification of steroid hormones in plasma using a surrogate calibrant approach and UHPLC-ESI-QTOF-MS/MS with SWATH-acquisition combined with untargeted profiling. Anal Chim Acta.

[109] Grebe SKG, Singh RJ (2016). Clinical peptide and protein quantification by mass spectrometry (MS). TrAC Trends Anal Chem.

[110] Kruve A, Kaupmees K, Liigand J, Leito I (2014). Negative electrospray ionization via deprotonation: predicting the ionization efficiency. Anal Chem.

[111] Yang J, Wen H, Zhang L, Zhang X, Fu Z, Li J (2017). The influence of ripening stage and region on the chemical compounds in mulberry fruits (*Morus atropurpurea* Roxb.) based on UPLC-QTOF-MS. Food Res Int..

[112] Sun X, Ouyang Y, Chu J, Yan J, Yu Y, Li X (2014). An in-advance stable isotope labeling strategy for relative analysis of multiple acidic plant hormones in sub-milligram *Arabidopsis thaliana* seedling and a single seed. J Chromatogr A.

[113] Erland LAE, Shukla MR, Glover WB, Saxena PK (2017). A simple and efficient method for analysis of plant growth regulators: a new tool in the chest to combat recalcitrance in plant tissue culture. Plant Cell Tissue Organ Cult PCTOC.

[114] Yoneyama K, Xie X, Sekimoto H, Takeuchi Y, Ogasawara S, Akiyama K (2008). Strigolactones, host recognition signals for root parasitic plants and arbuscular mycorrhizal fungi, from Fabaceae plants. New Phytol.

[115] Deng T, Wu D, Duan C, Guan Y (2016). Ultrasensitive quantification of endogenous brassinosteroids in milligram fresh plant with a quaternary ammonium derivatization reagent by pipette-tip solid-phase extraction coupled with ultra-high-performance liquid chromatography tandem mass spectrometry. J Chromatogr A.

[116] Cai WJ, Yu L, Wang W (2019). Simultaneous determination of multi-class phytohormones in sub-milligram plant samples by one-pot multi-functional derivatization assisted liquid chromatography-tandem mass spectrometry. Anal Chem..

[117] Liu Y, Fang XA, Chen G (2019). Recent development in sample preparation techniques for plant hormone analysis. TrAC Trends Anal Chem..

[118] Deng T, Wu D, Duan C, Yan X, Du Y, Zou J (2017). Spatial profiling of gibberellins in a single leaf based on microscale matrix solid-phase dispersion and precolumn derivatization coupled with ultraperformance liquid chromatography-tandem mass spectrometry. Anal Chem.

[119] Yu L, Ding J, Wang Y-L, Liu P, Feng Y-Q (2016). 4-Phenylaminomethyl-benzeneboric acid modified tip extraction for determination of brassinosteroids in plant tissues by stable isotope labeling-liquid chromatography–mass spectrometry. Anal Chem.

[120] Guo G, Li N (2011). Relative and accurate measurement of protein abundance using 15 N stable isotope labeling in Arabidopsis (SILIA). Phytochemistry.

[121] Liu J-F, Ding J, Yuan B-F, Feng Y-Q (2014). Magnetic solid phase extraction coupled with in situ derivatization for the highly sensitive determination of acidic phytohormones in rice leaves by UPLC-MS/MS. Analyst..

[122] Loba VC, Pollmann S, Kleine-Vehn J, Sauer M (2017). Highly sensitive salicylic acid quantification in milligram amounts of plant tissue. Plant Horm.

[123] Ayele BT, Magnus V, Mihaljević S, Prebeg T, Čož-Rakovac R, Ozga JA (2010). Endogenous gibberellin profile during christmas rose (*Helleborus niger* L.) flower and fruit development. J Plant Growth Regul..

[124] Shani E, Weinstain R, Zhang Y, Castillejo C, Kaiserli E, Chory J (2013). Gibberellins accumulate in the elongating endodermal cells of *Arabidopsis* root. Proc Natl Acad Sci.

[125] Ge L, Peh CYC, Yong JWH, Tan SN, Hua L, Ong ES (2007). Analyses of gibberellins by capillary electrophoresis–mass spectrometry combined with solid-phase extraction. J Chromatogr A.

[126] Ma L, Zhang H, Xu W, He X, Yang L, Luo Y (2013). Simultaneous determination of 15 plant growth regulators in bean sprout and tomato with liquid chromatography-triple quadrupole tandem mass spectrometry. Food Anal Methods.

[127] Zhang Z, Hao Y-H, Ding J, Xu S-N, Yuan B-F, Feng Y-Q (2015). One-pot preparation of a mixed-mode organic-silica hybrid monolithic capillary column and its application in determination of endogenous gibberellins in plant tissues. J Chromatogr A.

[128] Xin P, Yan J, Fan J, Chu J, Yan C (2013). An improved simplified high-sensitivity quantification method for determining brassinosteroids in different tissues of rice and *Arabidopsis*. Plant Physiol.

[129] Xin P, Yan J, Fan J, Chu J, Yan C (2013). A dual role of boronate affinity in high-sensitivity detection of vicinal diol brassinosteroids from sub-gram plant tissues via UPLC-MS/MS. Analyst..

[130] Chen M-L, Fu X-M, Liu J-Q, Ye T-T, Hou S-Y, Huang Y-Q (2012). Highly sensitive and quantitative profiling of acidic phytohormones using derivatization approach coupled with nano-LC–ESI-Q-TOF-MS analysis. J Chromatogr B.

[131] Zhang H, Tan SN, Teo CH, Yew YR, Ge L, Chen X (2015). Analysis of phytohormones in vermicompost using a novel combinative sample preparation strategy of ultrasound-assisted extraction and solid-phase extraction coupled with liquid chromatography–tandem mass spectrometry. Talanta.

[132] Liu Z, Cai B-D, Feng Y-Q (2012). Rapid determination of endogenous cytokinins in plant samples by combination of magnetic solid phase extraction with hydrophilic interaction chromatography–tandem mass spectrometry. J Chromatogr B.

[133] Farrow SC, Emery RN (2012). Concurrent profiling of indole-3-acetic acid, abscisic acid, and cytokinins and structurally related purines by high-performance-liquid chromatography tandem electrospray mass spectrometry. Plant Methods..

[134] An JH, Yuk HJ, Kim D-Y, Nho CW, Lee D, Ryu HW (2018). Evaluation of phytochemicals in Agastache rugosa (Fisch. & C.A.Mey.) Kuntze at different growth stages by UPLC-QTof-MS. Ind Crops Prod..

[135] Huang B-M, Zha Q-L, Chen T-B, Xiao S-Y, Xie Y, Luo P (2018). Discovery of markers for discriminating the age of cultivated ginseng by using UHPLC-QTOF/MS coupled with OPLS-DA. Phytomedicine.

[136] Jandrić Z, Roberts D, Rathor MN, Abrahim A, Islam M, Cannavan A (2014). Assessment of fruit juice authenticity using UPLC–QToF MS: a metabolomics approach. Food Chem.

[137] Bajguz A, Hayat S (2009). Effects of brassinosteroids on the plant responses to environmental stresses. Plant Physiol Biochem.

[138] Divi UK, Krishna P (2009). Brassinosteroid: a biotechnological target for enhancing crop yield and stress tolerance. New Biotechnol.

[139] Liu J, Zhang D, Sun X, Ding T, Lei B, Zhang C (2017). Structure-activity relationship of brassinosteroids and their agricultural practical usages. Steroids.

[140] Scaffidi A, Waters MT, Sun YK, Skelton BW, Dixon KW, Ghisalberti EL (2014). Strigolactone hormones and their stereoisomers signal through two related receptor proteins to induce different physiological responses in *Arabidopsis*. Plant Physiol.

[141] Chen M, Wang R, Zhu Y, Liu M, Zhu F, Xiao J (2018). 4-Mercaptophenylboronic acid-modified spirally-curved mesoporous silica nanofibers coupled with ultra performance liquid chromatography–mass spectrometry for determination of brassinosteroids in plants. Food Chem.

[142] Ćavar S, Zwanenburg B, Tarkowski P (2015). Strigolactones: occurrence, structure, and biological activity in the rhizosphere. Phytochem Rev.

[143] Foo E, Davies NW (2011). Strigolactones promote nodulation in pea. Planta.

[144] Kohlen W, Charnikhova T, Liu Q, Bours R, Domagalska MA, Beguerie S (2011). Strigolactones are transported through the xylem and play a key role in shoot architectural response to phosphate deficiency in nonarbuscular mycorrhizal host *Arabidopsis*. Plant Physiol.

[145] Zhang Q, Zhou L, Chen H (2016). Solid-phase microextraction technology for in vitro and in vivo metabolite analysis. Trends Anal Chem..

[146] Nemes P, Vertes A (2012). Ambient mass spectrometry for in vivo local analysis and in situ molecular tissue imaging.Trends. Anal Chem..

[147] Kou X, Chen G (2019). In vivo sampling: a promising technique for detecting and profiling endogenous substances in living systems. J Agr Food Chem..

[148] Xia L, Yang J (2020). Recent progress in fast sample preparation techniques. Anal Chem..

